# Multiobjective Emergency Resource Allocation under the Natural Disaster Chain with Path Planning

**DOI:** 10.3390/ijerph19137876

**Published:** 2022-06-27

**Authors:** Feiyue Wang, Ziling Xie, Hui Liu, Zhongwei Pei, Dingli Liu

**Affiliations:** 1 Institute of Disaster Prevention Science and Safety Technology, School of Civil Engineering, Central South University, Changsha 410075, China; wfycsu@csu.edu.cn (F.W.); 204811137@csu.edu.cn (Z.X.); liuhui0421@csu.edu.cn (H.L.); 2 School of Resources and Safety Engineering, Central South University, Changsha 410083, China; 3 Department of Engineering Management, Changsha University of Science and Technology, Changsha 410114, China

**Keywords:** natural disaster chain, emergency resource allocation, multiobjective optimization, path planning

## Abstract

Public safety and health cannot be secured without the comprehensive recognition of characteristics and reliable emergency response schemes under the disaster chain. Distinct from emergency resource allocation that focuses primarily on a single disaster, dynamic response, periodic supply, and assisted decision-making are necessary. Therefore, we propose a multiobjective emergency resource allocation model considering uncertainty under the natural disaster chain. Resource allocation was creatively combined with path planning through the proposed multiobjective cellular genetic algorithm (MOCGA) and the improved A* algorithm with avoidance of unexpected road elements. Furthermore, timeliness, efficiency, and fairness in actual rescue were optimized by MOCGA. The visualization of emergency trips and intelligent avoidance of risk areas were achieved by the improved A* algorithm. The effects of logistics performance, coupling of disaster factors, and government regulation on emergency resource allocation were discussed based on different disaster chain scenarios. The results show that disruption in infrastructure support, cascading effect of disasters, and time urgency are additional environmental challenges. The proposed model and algorithm work in obtaining the optimal solution for potential regional coordination and resilient supply, with a 22.2% increase in the total supply rate. Cooperative allocation complemented by political regulation can be a positive action for successfully responding to disaster chains.

## 1. Introduction

Public safety and health have become prominent livelihood issues nowadays. China’s 14th five-year development plan clearly suggests that the evolution mechanism and intelligent control theory of multidisaster coupling are important fields for priority development [1]. With the intensification in the suddenness, abnormality, and cascading destruction of natural disasters, the consequences of the disaster chain are significantly costly [2]. This phenomenon has attracted extensive attention from the international community. For example, both the Mekong Countries symposium and UK’s New Dimensions program have been focusing on improving joint operational capacity in response to multiple disasters [3,4]. The allocation of emergency resources plays a crucial role in social stability and life maintenance when the unexpected disaster chain occurs. In response to the disaster chain, there is an urgent need to establish a new emergency resource allocation pattern that can realize the linkage of resource sharing, coordination, and efficiency.

Previous studies have mainly concentrated on geologic genesis and multihazard framework of disaster chains [5,6]. Scholars in different fields have studied emergency resource allocation problems. Aalami and Kattan developed a method for resource allocation to communities during evacuation processes and compared four problem variations [7]. Cavdur et al. developed a two-stage stochastic integer programming model with the first stage assigning facilities and the second stage distributing supplies [8]. Sheu and Pan integrated shelter network, medical network, and distribution network to support emergency logistics operations in response to large-scale natural disasters [9]. Nevertheless, most of the previous studies have been devoted to one-time disaster attacks [10]. Secondary disasters, especially the resources competition and uncertainty caused by dramatic derivation, have been ignored. Furthermore, the uncertainty in supply, delivery trips, and disaster evolution need to be considered in allocation decisions.

Moreover, only one objective function has been considered in most previous studies. Liu et al. addressed the problem of emergency medical vehicle allocation in urban areas to maximize the coverage of demand sites [11]. Other emergency resource scheduling problems of single objective have been proposed and discussed [12,13,14,15]. However, single-objective methods have a limitation that may severely restrict rescue operations. The application of multiobjective approaches is scientific because actual emergency response generally requires taking into account multiple operational objectives, such as response time, resource efficiency, and supply fairness. Besides, the emergency resource allocation problem is a Nondeterministic Polynomial-hard (NP-hard) problem. One of the widely used solutions is to transform the multiobjective function into the single-objective function [16,17]. Due to the lack of a convincing basis for objective parameters, this transformation faces some problems when carried out. The method to overcome this drawback is to implement parallel optimization via heuristics [18]. Heuristic algorithms have been widely adopted in logistics problems, such as ant colony optimization, tabu search algorithm, and genetic algorithm [19,20,21,22].

The reliability and time costs of transportation paths are very important due to the urgency of resource availability after a disaster [23]. Distance is commonly measured by Euclidean distance and Manhattan distance [24,25,26]. For emergency travel time, most studies have been derived from Global Information System or estimated from scenario settings [27,28,29,30]. However, little research has been done on the problems arising from the performance of dynamic road networks, and there is a research gap of combining resource allocation with path planning. The A* algorithm and its modifications are the most classical heuristic pathfinding methods and are applied to path planning for workshop robots [31]. In most cases, searching for the optimal path is equivalent to identifying the least costly or fastest path [32]. However, the limitations of traditional pathfinding are highlighted by the single pathfinding result, low intelligence, and large gap with manual selection. The traditional A* algorithm can only avoid static obstacles, while a dynamic anti-collision A* algorithm is proposed to solve the path planning problem with dynamic obstacles in multiship encounter scenarios [33]. The simulation results show that the dynamic A* algorithm can generate more reasonable dynamic and static obstacle avoidance paths in complex traveling scenarios. The A* algorithm can be further improved so that path planning can be properly integrated into emergency planning.

The framework for disaster risk reduction sets out priority objectives for managing disaster risk and ultimately enhancing preparedness for effective response [34]. Considering the relationship between society and environment, strategies to improve risk acceptance and to motivate transformation in the context of changing disasters are proposed [35]. The key to disaster risk reduction is to reduce the exposure and vulnerability of people and property to hazards without compromising the long-term prospects of communities or countries. A critical component of disaster risk reduction is demand-led multidisaster resilience [36]. However, few works have realized the key to save lives in disasters, i.e., the ability to sustain relief [37]. Therefore, there is a significant need to reduce the risk of the natural disaster chain through multiperiod emergency resource allocation.

In light of the above considerations, a multiobjective emergency resource allocation model with path planning is proposed to be applied for the natural disaster chain in this study. From the results of this paper, the model performed well in dynamic decision, periodic supply, and cascading response. Although the A* algorithm has been widely used in many other fields, the provided perspective can be valuable in application for emergency management. The multiobjective cellular genetic algorithm (MOCGA) was applied to realize timeliness, effectiveness, and fairness of relief process and the A* algorithm was improved to support path planning in the multiechelon road network.

The main contributions of this study are as follows: Firstly, an emergency resource allocation model that integrates the multiobjective, multiperiod, and multiechelon network is developed for the natural disaster chain under the uncertainties. Secondly, the interconnection of the improved A* algorithm and the MOCGA makes dynamic response and periodic supply feasible. Thirdly, through a series of numerical study, the key points of resource allocation in actual emergency relief are analyzed. The remainder of this paper is organized as follows: The formulation of the proposed model and improved path planning are described in Section 2. The multiobjective cellular genetic algorithm is presented in Section 3. The numerical study of the three scenarios is analyzed in Section 4. In Section 5, the conclusions and future scopes related to this work are given.

## 2. Problem Formulation

### 2.1. Problem Description

This paper concentrates on the multiperiod and multiobjective problem of emergency resource allocation for the natural disaster chain, with the vehicle path problem for multiechelon logistics networks. Emergency resource allocation under the natural disaster chain is complex and often accompanied with uncertainty and conflicting objectives. In order to respond rapidly to the disaster chain, decision makers need better technical support and well-organized deployment of facilities. Responsiveness refers to distributing the maximum supplies to the maximum number of beneficiaries at the right time [38]. Generally, national resource reserves are strategically placed throughout the country to cover multiple disasters. Owing to the derivative scope and social impact of the natural disaster chain, it is far from enough to depend on local relief facilities alone. Warehouses can serve as cross-regional relief facilities and provide stored emergency resources for rescue stations, which are closer to disaster sites. In addition, rescue stations play the role of integrating transported resources and redistributing them to the corresponding disaster sites. The disaster sites can be classified into primary and secondary ones according to the cause of their outbreak.

Given the time-dependent demand, generic and specific resources need to be transported from multiple warehouses to each disaster site. The time, location, type, and quantity of allocated resources must be given throughout the response phase. The purpose of this study is to develop a dynamic decision process and periodic supply strategy for the challenging problem. It is considered as a multiobjective model for emergency resource allocation under the natural disaster chain. A three-echelon emergency logistics network is constructed, consisting of warehouses (WHs), rescue stations (RSs), primary disaster points (PDPs), and secondary disaster points (SDPs).

Although secondary disasters are induced by primary disasters in the chain structure, the disaster types and required emergency resources often differ. For example, the natural disaster chain of landslide-induced surge is a widespread form in mountainous and gorge areas, coastal areas, and reservoir areas [39]. Related secondary disasters resulting from the consequences of strong earthquake damage include landslides, floods, mudslides, fires, and pollution, etc. The need for generic and specific resources in response to the disaster chain makes the trade-off difficult. The uncertainty arises from the disaster derivation and information asymmetry in the subsequent decision. Thereby, fuzzy sets are employed to represent the fluctuation intervals in the supply and demand. The single transportation mode is considered, and the travel time is derived from planned paths on the raster map, which is in relation to the distance and road conditions. Moreover, the recirculation of resource flows is avoided in the logistics network.

- Sets.

I: Set of WH, indexed by i.

J: Set of RS, indexed by j.

K: Set of PDP, indexed by k.

S: Set of SDP, indexed by s.

T: Set of time periods, indexed by t.

H: Set of emergency resource types, indexed by h.

- Parameters.

$\tilde{G_{i}^{h,t}}$: The quantity of emergency resources h supplied by WH i in time period t.

$\tilde{D_{k}^{h,t}}$: The quantity of emergency resources h PDP k demands in time period t.

$\tilde{D_{s}^{h,t}}$: The quantity of emergency resources h SDP s demands in time period t.

$d_{i,j}$: The distance between WH i and RS j.

$d_{j,k}$: The distance between RS j and PDP k.

$d_{j,s}$: The distance between RS j and SDP s.

$p_{s}$: The probability of secondary disaster at SDP s.

- Variables.

$x_{i,j}^{h,t}$: The quantity of emergency resources h from WH i to RS j in time period t. $x_{i,j}^{h,t}\in N$.

$x_{j,k}^{h,t}$: The quantity of emergency resources h from RS j to PDP k in time period t. $x_{j,k}^{h,t}\in N$.

$x_{j,s}^{h,t}$: The quantity of emergency resources h from RS j to SDP s in time period t. $x_{j,s}^{h,t}\in N$.

$U_{k}^{h,t}$: The quantity of unsatisfied demand h of PDP k in time period t. $U_{k}^{h,t}\in N$.

$U_{s}^{h,t}$: The quantity of unsatisfied demand h of PDP s in time period t. $U_{s}^{h,t}\in N$.

### 2.2. Defuzzification

The allocation of emergency resources under the natural disaster chain is noted as inherently difficult and constitutes a spatiotemporal decision under uncertainty. As a method to describe uncertainty phenomena, fuzzy sets are frequently used in production and life, such as fuzzy control systems and fuzzy classification [40,41,42]. In order to reflect uncertainty in the rescue process of the disaster chain, the triangular fuzzy number $\tilde{G_{i}^{h,t}},\;\tilde{D_{k}^{h,t}},\;\tilde{D_{s}^{h,t}}$ and the subsequent defuzzification approach are employed [43]. Take $\tilde{G_{i}^{h,t}}$ as an example, $\tilde{G_{i}^{h,t}}=\left({\left[G_{i}^{h,t}\right]}^{1},{\left[G_{i}^{h,t}\right]}^{2},{\left[G_{i}^{h,t}\right]}^{3}\right)$, where ${\left[G_{i}^{h,t}\right]}^{1}$, ${\left[G_{i}^{h,t}\right]}^{2}$, ${\left[G_{i}^{h,t}\right]}^{3}$ are the pessimistic, normal, and optimistic values of a fuzzy number, respectively. The conversion is then executed according to the weighted method, $\tilde{G_{i}^{h,t}}=\left({\left[G_{i}^{h,t}\right]}^{1},{\left[G_{i}^{h,t}\right]}^{2},{\left[G_{i}^{h,t}\right]}^{3}\right)=\omega_{1}{\left[G_{i}^{h,t}\right]}^{1}+\omega_{2}{\left[G_{i}^{h,t}\right]}^{2}+\omega_{3}{\left[G_{i}^{h,t}\right]}^{3}$, where $\omega_{1}+\omega_{2}+\omega_{3}=1$. In essence, the values of pessimistic, normal, and optimistic situations are estimated from years of disaster data.

### 2.3. Path Planning with Improved A* Algorithm

Path planning can support allocation decisions to avoid risky road sections and obtain optimal distribution trips and corresponding travel times. The regional road network is at risk of disaster chain disruption. It is necessary to optimize the timeliness and reliability of emergency resource transportation to ensure resources can safely reach primary and potential secondary disaster sites in time. From the actual disaster disposal, logistics access scenarios should be set up to reflect potential road interruptions and blockages.

The A* algorithm is organically combined with the raster map and road reliability is added to the traditional shortest path search. The purpose of path planning is to find the optimal path that meets the practical requirements from the starting nodes to the target nodes in the road network. The logistics network is abstracted as a directed connectivity graph G=(V,R,P)*,* where V is the set of nodes, R is the set of connection arcs between nodes, and $P=\left\{P_{c}|c=1,2,\ldots ,C\right\}$ is the set of arc resistances.

Maps with arbitrary contours can be drawn with sufficiently fine raster to simulate the real road environment. Each raster is colored differently to characterize the specific physical entity. There are three directions for path planning based on raster maps: horizontal, vertical, and diagonal. For low-speed robots, the planned path can be followed exactly, while for medium- and high-speed robots, the planned path can be smoothed for non-fully-constrained systems.

**Figure 1:**
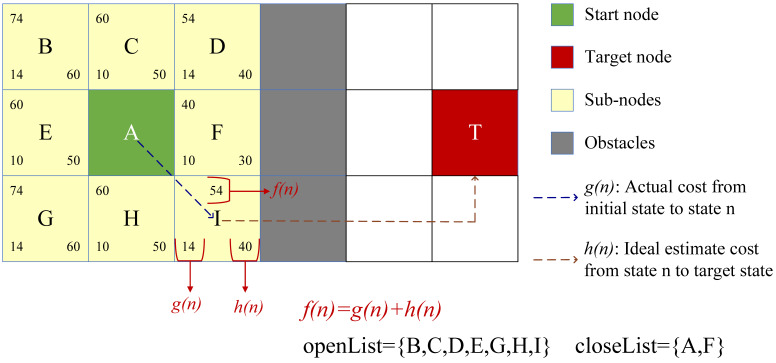
A* algorithm evaluation function search.

Based on the estimated cost, A* algorithm iterates from the initial state through state *n* to the target state. The search process of the evaluation function is shown in Figure 1, where g(n) is the actual cost from the initial state to state *n* in the state space, representing the information blocks near the start node; h(n) is the ideal estimated cost from state *n* to the target state, representing the information blocks near the target node. The total cost f(n) equals the sum of g(n) and h(n), f(n)=g(n)+h(n). Two list sets are defined: openList and closeList. The movement cost f(n) of the subnodes around the start node is evaluated and the node with the smallest cost is selected and added to the closeList. This operation is repeated in the openList and the parent node is updated. Once the target node is searched, the path search is completed.

Based on the traditional shortest path planning, the A* algorithm is modified to overcome the poor flexibility and low applicability of single-objective pathfinding, which can only avoid static obstacles. The improved algorithm for the avoidance of random obstacles can simulate the unexpected road elements that may occur during the allocation of emergency resources, such as road disruption and blockage. Two main planning objectives are concerned, reflecting the timeliness and reliability of the trips. Trip efficiency refers to optimizing the actual travel time of the distribution vehicle, measured by the raster from start nodes to target nodes. Trip reliability refers to avoiding risky road sections and reducing trip exposure. Road section reliability is translated into a safety factor for each raster.

### 2.4. Multiobjective Emergency Resource Allocation Model

The problem of emergency resource allocation is modeled to obtain a robust and scientific scheme to the disaster chain. The social and humanitarian attributes of the disaster chain relief dictate that the process is inevitably accompanied by multiple conflicting objectives. As an essential evaluation index of rescue schemes, timeliness determines whether the disaster sites can be allocated in time. According to the consensus of the rescue community, the golden relief time after a natural disaster is 72 h, after which the survival rate decreases significantly. It is necessary to avoid delays in the availability of resources. Furthermore, the complexity of the rescue network and the diversity of resource demand make it difficult to decide on a reasonable allocation solution. In the process of emergency resource allocation in the natural disaster chain, the demand comes from the primary disaster points and secondary disaster points, which is reflected in the types and quantity. Emergency resource allocation is a special logistics activity to scientifically distribute emergency resources from the warehouses to each disaster area. However, the supply of emergency resources from the warehouses is often limited at the initial stage. The excessive influx of emergency resources can result in wasted supplies, while insufficient resources can lead to further casualties and losses. Consequently, delivery time and unsatisfied demand are taken as multiple objectives for the model.

Different from the commercial logistics problem, the proposed emergency resource allocation emphasizes both the timeliness and the effectiveness of schemes. The optimal allocation scheme is in compromise between different evaluation indexes. The multiobjective model for emergency resource allocation is proposed as follows:

(1) $$Min\;\sum_{t\in T}\sum_{i\in I}\sum_{j\in J}\sum_{h\in H}x_{i,j}^{h,t}d_{i,j}+\sum_{t\in T}\sum_{j\in J}\sum_{k\in K}\sum_{h\in H}x_{j,k}^{h,t}d_{j,k}\;+\sum_{t\in T}\sum_{j\in J}\sum_{s\in S}\sum_{h\in H}p_{s}x_{j,s}^{h,t}d_{j,s}$$

(2) $$Min\;\sum_{t\in T}\sum_{k\in K}\sum_{h\in H}U_{k}^{h,t}+\sum_{t\in T}\sum_{s\in S}\sum_{h\in H}U_{s}^{h,t}$$

Subject to

(3) $$x_{i,j}^{h,t},\;x_{j,k}^{h,t},\;x_{j,s}^{h,t}\geq 0,\;i\in I,j\in J,k\in K,s\in S,h\in H,t\in T$$

(4) $$\sum_{t\in T}\sum_{j\in J}x_{i,j}^{h,t}\leq \tilde{G_{i}^{h,t}},j\in J,t\in T,h\in H$$

(5) $$U_{k}^{h,1}=\tilde{D_{k}^{h,1}}-\sum_{j\in J}x_{j,k}^{h,1},k\in K,h\in H$$

(6) $$U_{k}^{h,t}=\tilde{D_{k}^{h,t}}-\sum_{j\in J}x_{j,k}^{h,t}+U_{k}^{h,t-1},k\in K,h\in H,t\in T,t\geq 2$$

(7) $$U_{s}^{h,1}=\tilde{D_{s}^{h,1}}-\sum_{j\in J}x_{j,s}^{h,1},s\in S,h\in H$$

(8) $$U_{s}^{h,t}=\tilde{D_{s}^{h,t}}-\sum_{j\in J}x_{j,s}^{h,t}+U_{s}^{h,t-1},s\in S,h\in H,t\in T,t\geq 2$$

(9) $$\sum_{j\in J}x_{j,k}^{h,1}\leq \tilde{D_{k}^{h,1}},k\in K,h\in H$$

(10) $$\sum_{j\in J}x_{j,k}^{h,t}\leq \tilde{D_{k}^{h,t}}+U_{k}^{h,t-1},k\in K,h\in H,t\in T,t\geq 2$$

(11) $$\sum_{j\in J}x_{j,s}^{h,1}\leq \tilde{D_{s}^{h,1}},s\in S,h\in H$$

(12) $$\sum_{j\in J}x_{j,s}^{h,t}\leq \tilde{D_{s}^{h,t}}+U_{s}^{h,t-1},s\in S,h\in H,t\in T,t\geq 2$$

(13) $$\sum_{i\in I}x_{i,j}^{h,t}\geq \sum_{k\in K}x_{j,k}^{h,t}+\sum_{s\in S}x_{j,s}^{h,t},h\in H,j\in J,t\in T$$

Equation (1) represents the timeliness of the model, labeled as F1, with the aim of minimizing the emergency travel time, which is related to transported resources and road conditions among sites. Equation (2) represents the efficiency of the model, labeled as F2, with the aim of minimizing the unsatisfied demand from PDPs and SDPs, which is related to the amount of supplied resources. Equations (3)–(13) are the feasibility constraints. Considering the availability of realistic assumptions, the transportation flow is ensured to be unidirectional by Equation (3). The quantity of transported resources is limited to the supply of WHs by Equation (4). Equations (5)–(8) are used to determine the quantity of unsatisfied demand of PDPs and SDPs at each period. Equations (9)–(12) are used to limit the quantity of resources to PDPs and SDPs no more than their demand at each period. The quantity of resources to PDPs and SDPs is no more than the resources available to RSs, as shown in Equation (13).

## 3. Multiobjective Cellular Genetic Algorithm

**Figure 2:**
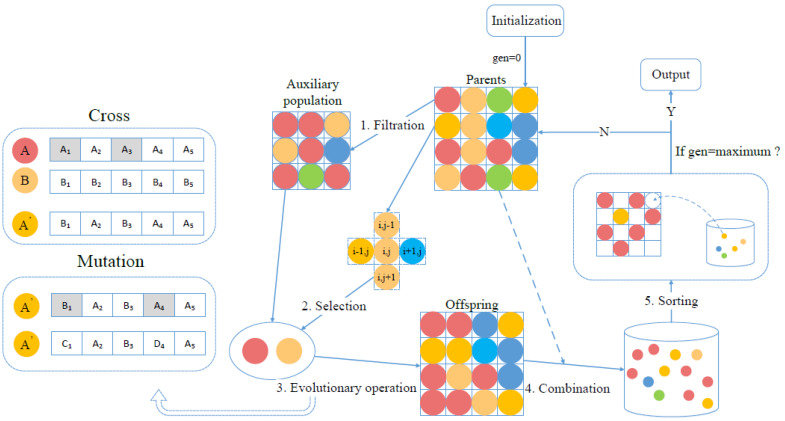
The structure and procedure of MOCGA.

The emergency resource allocation problem is NP-hard and many heuristic algorithms have been used to solve this problem. Compared to other algorithms, MOCGA is equipped with the domain structure and evolutionary mechanism to achieve the balance between local and global optimization [44,45]. The quality and diversity of the optimal solution set at convergence are guaranteed. Its excellent optimization performance has led to its application in many fields [46,47,48]. Thus, MOCGA is chosen to optimize the multiobjective model for emergency resource allocation to provide comprehensive and valuable schemes. The flowchart of MOCGA is shown in Figure 2.

### 3.1. Chromosome Coding and Initialization

Real number coding is adopted because it can reflect resource flow during the rescue process. Each chromosome of MOCGA determines an allocation scheme and indicates the emergency resource flows between WHs, RSs, PDPs, and SDPs. Thus, each chromosome in period t is represented as $C_{t}=\left\{C_{t}^{1},C_{t}^{2}\right\}$, where the former part $C_{t}^{1}$ determines the resources for RSs, $C_{t}^{1}=\left(x_{1,1}^{1,t}\cdots x_{I,1}^{1,t}\cdots x_{I,J}^{1,t}\cdots x_{I,J}^{H,t}\right)$, and the latter part $C_{t}^{2}$ determines the resources for PDPs and SDPs, $C_{t}^{2}=\{\left(x_{1,1}^{1,t}\cdots x_{J,1}^{1,t}\cdots x_{J,K}^{1,t}\cdots x_{J,K}^{H,t}\right)\},\left(x_{1,1}^{1,t}\cdots x_{J,1}^{1,t}\cdots x_{J,S}^{1,t}\cdots x_{J,S}^{H,t}\right)\}$.

The chromosomes are initialized randomly to ensure the diversity. Constraints are attached to make chromosomes comply with the actual conditions. When the former part $C_{t}^{1}$ is initialized, $x_{1,1}^{h,t}$ is generated in the range $\left(0,\tilde{G_{i}^{h,t}}\right)$ and $x_{i,j}^{h,t}$ is generated in the range $\left(0,\tilde{G_{i}^{h,t}}-\overset{j-1}{\underset{j=1}{\sum }}x_{i,j}^{h,t}\right)$. When $\overset{j}{\underset{j=1}{\sum }}x_{i,j}^{h,t}>\tilde{G_{i}^{h,t}}$, $x_{i,j}^{h,t}=0$; if it does not match the constraints, it will be set to 0. When the latter part $C_{t}^{2}$ is initialized, $x_{j,k}^{h,t}$ is generated in the range $\left(0,\underset{i\in I}{\sum }x_{i,j}^{h,t}-\overset{k-1}{\underset{k=1}{\sum }}x_{j,k}^{h,t}\right)$ and it will be set to 0 if $\overset{k}{\underset{k=1}{\sum }}x_{j,k}^{h,t}>\underset{i\in I}{\sum }x_{i,j}^{h,t}$. $x_{j,s}^{h,t}$ is generated in the range $\left(0,\underset{i\in I}{\sum }x_{i,j}^{h,t}-\underset{k\in K}{\sum }x_{j,k}^{h,t}-\overset{s-1}{\underset{s=1}{\sum }}x_{j,s}^{h,t}\right)$ and it will be set to 0 if $\overset{s-1}{\underset{s=1}{\sum }}x_{j,s}^{h,t}>\underset{i\in I}{\sum }x_{i,j}^{h,t}-\underset{k\in K}{\sum }x_{j,k}^{h,t}$.

### 3.2. Fitness Evaluation and Selection

In order to evaluate the fitness for multiobjective optimization, the chromosomes undergo rapid nondominated sorting [49]. The fitness of chromosome Fitness(C) is defined according to the layer and crowding distance. The chromosomes with lower fitness are considered to be superior.

There are two types of selection in the algorithm iteration. The first one is for the evolutionary operation. In the two-dimensional grids, the selection is conducted within the set π, which consists of the central cell ${\left(C_{t}\right)}_{i,j}$ and its neighborhood structure. The selection probability is represented as $P_{i,j}=Fitness{\left(C_{t}\right)}_{i,j}/\underset{x,y\in \pi }{\sum }Fitness{\left(C_{t}\right)}_{x,y}$. It differs from the genetic algorithm in that the operation is restricted to the set π and diverse populations. The other is to select the optimal chromosome from the Pareto front θ for the next period of optimization, as shown in the following equations [50]:

(14) $$\alpha_{i}=\{\begin{array}{c} \begin{array}{cc} 1 & F_{i}\leq \end{array}F_{i}^{min}\\ \begin{array}{cc} \frac{F_{i}^{max}-F_{i}}{F_{i}^{max}-F_{i}^{min}} & F_{i}^{min}<F_{i}<F_{i}^{max} \end{array}\\ \begin{array}{cc} 0 & F_{i}\geq \end{array}F_{i}^{max} \end{array},\;i\in N.\;$$

(15) $$\alpha \left(m\right)=\frac{\sum_{i\in N}\alpha_{i}\left(m\right)}{\sum_{m\in \theta }\sum_{j\in N}\alpha_{j}\left(m\right)}$$

where N is the number of objective functions, $F_{i}$ is the value of the objective function i, and $F_{i}^{max}$ and $F_{i}^{min}$ represent the minimum and maximum value of $F_{i}$ in the Pareto front θ.

### 3.3. Constraints Handling

Since the proposed emergency resource allocation model is a constrained one, it is critical that the optimal scheme obtained is feasible. Accordingly, the evolutionary process is subject with constraints handling so that the generated chromosome $C_{t}{}^{*}$ is feasible. If evolutionary operation is conducted in the resource flow between WHs and RSs, $\underset{c\in \left(K\cup S\right)}{\sum }x_{b,c}^{h,t}-\underset{i\in I,i\neq a}{\sum }x_{i,b}^{h,t}\leq x_{a,b}^{h,t}{}^{*}\leq \tilde{G_{a}^{h,t}}-\underset{j\in J,j\neq b}{\sum }x_{a,j}^{h,t}$. It maintains a balance between resource flow out of WHs and into RSs. If the evolutionary operation is conducted in the resource flow between RSs and PDPs or SDPs, $\tilde{D_{b}^{h,t}}-\underset{c\in J,c\neq a}{\sum }x_{c,b}^{h,t}\leq x_{a,b}^{h,t}{}^{*}\leq \underset{i\in I}{\sum }x_{i,a}^{h,t}-\underset{c\in \left(K\cup^{\;}S\right),c\neq b}{\sum }x_{a,c}^{h,t}$. It maintains the balance between resource flows out of RSs and into PDPs or SDPs.

## 4. Numerical Study

In connection with the actual situation of the disaster chain, the in-depth study of the emergency resource allocation process is a prerequisite for effective control of catastrophe risks. Logistics network is an important element of emergency relief. The research trend on disaster chain is associated with the dynamic simulation of the cumulative amplification effect. Government regulation is the ultimate attribution of disaster management. To verify whether the above elements play a positive or negative role in disaster chain system, three numerical scenarios related to logistics performance, coupling of disaster factors, and government regulation were analyzed. The main practical problems faced in emergency response of the disaster chain were identified, and theoretical support and effective measures were explored.

### 4.1. Road Network Visualization

**Figure 3:**
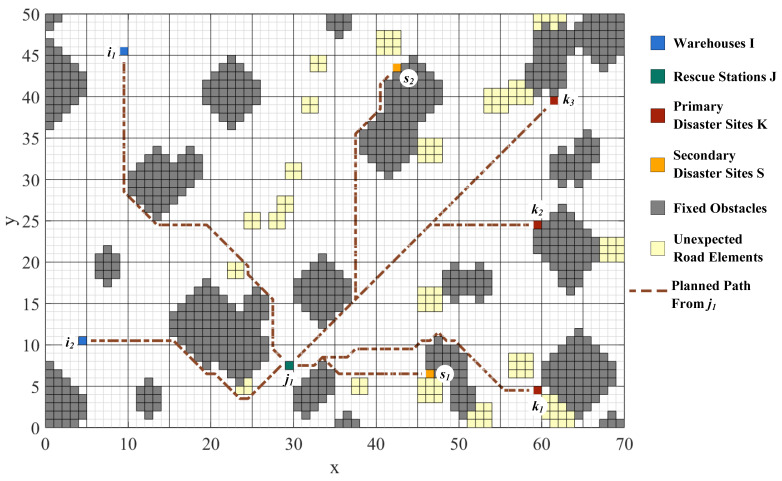
Paths from Rescue Station $j_{1}$ to each site.

**Figure 4:**
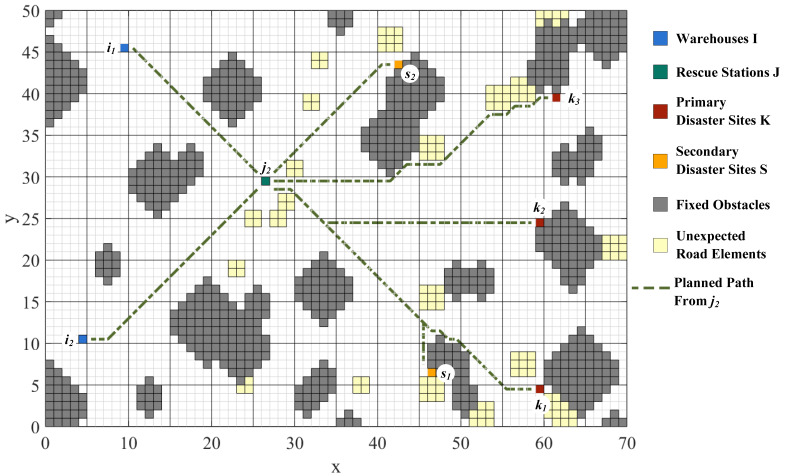
Paths from Rescue Station $j_{2}$ to each site.

**Figure 5:**
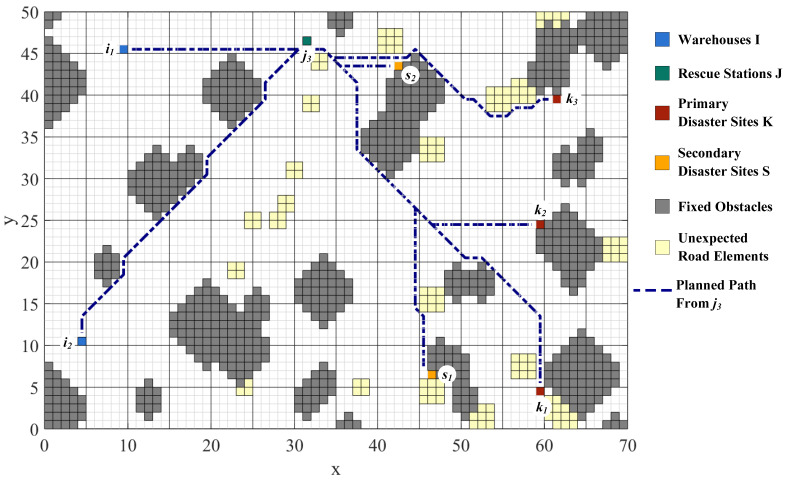
Paths from Rescue Station $j_{3}$ to each site.

**Figure 6:**
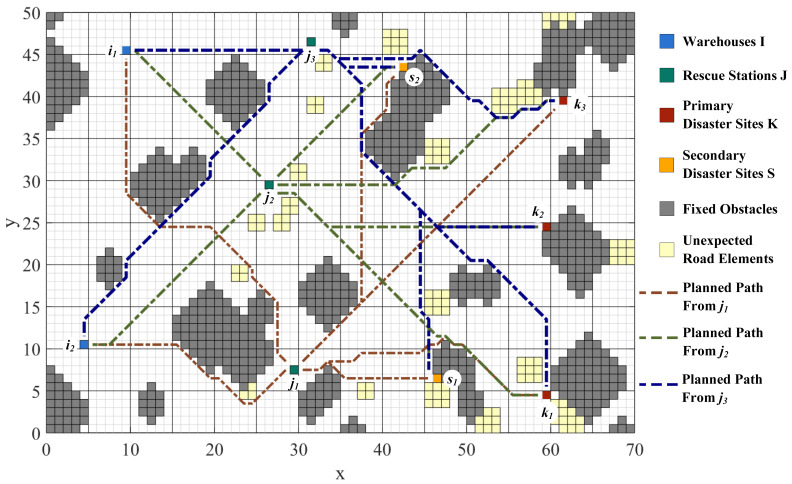
Paths’ Overview.

Road network construction and path planning are performed by means of the improved A* algorithm with the raster map. To model the practical road network, the grids represent different physical entities. Green grids represent rescue stations, red grids represent primary disaster points, yellow grids represent secondary disaster points, blue grids represent warehouses, gray grids represent fixed obstacles, and light-yellow grids represent unexpected road elements. It enables visualization of paths among points and the flexibility to avoid unexpected road conditions. The planned paths from Rescue Station $j_{1}$, $j_{2}$, and $j_{3}$ to each site and the paths’ overview are shown in Figure 3, Figure 4, Figure 5 and Figure 6.

The distances among WHs, RSs, PDPs, and SDPs are determined based on the planning results, as shown in Table 1. At the same time, the important nodes in the road network can be analyzed from the path generation, providing theoretical support for the reliability of emergency transportation.

### 4.2. Scenarios Design and Study

The actual disaster chain fluctuates due to risk environment and system factors. The proposed model and algorithm are validated with numerical scenarios. The literature research is followed to summarize some key perspectives of general interest nowadays. Three scenarios related to disaster relief operations are included, i.e., logistics performance, coupling of disaster factors, and government regulation.

#### 4.2.1. Parameters and Data

In the algorithm’s preselection, it is found that the Pareto front of MOCGA fully dominates the nondominated sorting genetic algorithm (NSGA-II). The algorithm parameters of MOCGA are set as follows: The area of cell space is set to 12 × 10, the population size is set to 120, the maximum number of iterations is set to 500, the probability of crossover $p_{c}$ is set to 0.6, and mutation $p_{m}$ is set to 0.9. The probability of secondary disasters in each site is randomly set to (0.7, 0.5). The supply of WHs and the demand from PDPs and SDPs are shown in Table 2 and Table 3, which can be generated by the population of disaster sites. In reality, the probability of secondary disasters, supply, and demand can be estimated based on rescue records over the years.

#### 4.2.2. Logistics Performance of the Hierarchical Network

**Figure 7:**
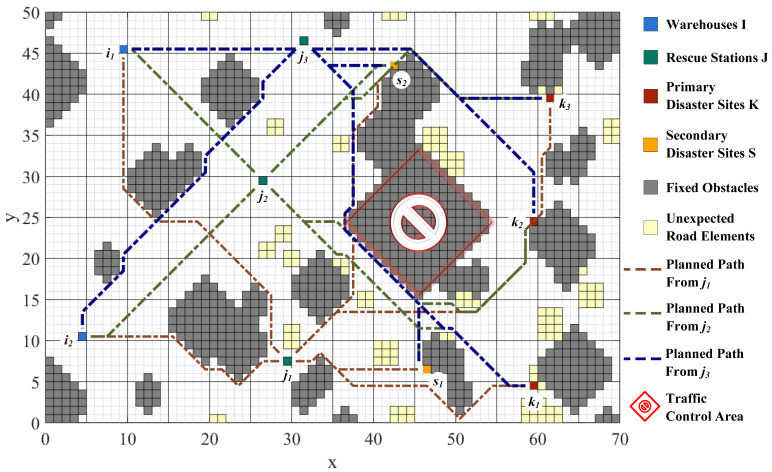
Detours when the key transportation hub fails.

This section investigates the influence of logistics access performance on the objective functions. Through path planning, a specific area in the central–eastern part of the map is identified as having the highest traffic load. The transportation road network with the collapsed key hub is shown in Figure 7. It can be noted that detours become inevitable. The disrupted multiechelon logistics network poses a challenge to emergency transit activities.

**Figure 8:**
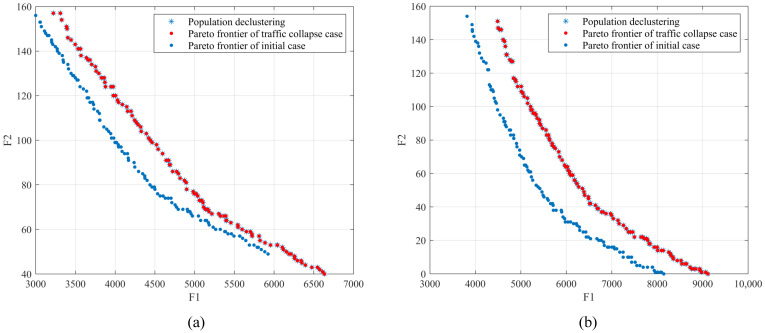
Population scatter comparison of the secondary disaster in period (**a**) $T_{1}$ and (**b**) $T_{2}$.

The breakdown of the transportation hub results in an overall suboptimal allocation of emergency resources, both in terms of unsatisfied demand and the time cost. A comparison of the population scatter in period T=1 and period T=2 is shown in Figure 8a,b. The Pareto front of normal road network dominates the distribution of detour. The unmet demand increases by 16.1%, yet delivery time is only 1.4% lower than the original base.

Notably, it validates the environmental challenges raised through semistructured interviews with disaster management agencies [51]—that is, unexpected events, disruptions in infrastructure support, cascading effect of disasters, and time urgency become additional environmental pressures on relief actions. Pre-emptive maintenance of critical infrastructure serves better than temporary rerouting on wartime. It suggests that the development of resilient infrastructure, with a shift from remediation to preparedness, will greatly contribute to withstanding the effects of environmental damage.

#### 4.2.3. Coupling Effect of Disaster Chain Factors

The overall disaster situation is often not a simple superposition of several individual disasters [52]. The disaster chain is a complex disaster system composed of the causative factors chain, gestation environment, and the disaster-bearing body. A disaster situation is formed by the complicated coupling in time and space of characteristics, such as the hazard of causative factors, the instability of gestation environment, and the vulnerability of the disaster-bearing body.

It is found that the gestation environment in a disaster chain is often modified by the preceding disaster type. With different hazards in the natural disaster chain, the vulnerability and exposure of the disaster-bearing body are not simply linear superposition. The causative factors chain formed by the interaction among hazards is often constrained by the gestation environment. The feedback effects of the disaster-bearing body on the causative factors chain are complex and it can be transformed into a causative factor under some conditions. The triggering of causative factors chain makes it possible for the disaster-bearing body to be subject to multiple forms of attacks, resulting in the concurrent and cascading phenomenon with disasters’ cumulative amplification.

This section describes the challenges posed by a disaster’s cumulative amplification on the allocation of emergency resources. Due to disaster-bearing properties, $S_{1}$ is sensitive to the disaster propagation of $K_{1}$. $K_{2}$ is located in the middle of $K_{1}$ and $K_{3}$, and its gestation environment changes subsequently. Based on the analysis of disaster cases, the typical characteristics of the natural disaster chain are classified as cumulative action type, short-term action type, and dynamic action type. The demand of $S_{1}$ and $K_{2}$ changes at a particular time, which in turn changes the supply and demand. Accordingly, it is assumed that the demand of $S_{1}$ changes in the second period and the demand of $K_{2}$ changes in the first period.

Observing the initial relief capacity, the supply/demand ratio $R_{T_{1}}^{H_{1}}$ of resource $H_{1}$ in period $T_{1}$ is 85.7%. The ratio $R_{T_{1}}^{H_{2}}$ of resource $H_{2}$ in period $T_{1}$ is 75.0%. The ratio $R_{T_{2}}^{H_{1}}$ of resource $H_{1}$ in period $T_{2}$ is 91.3%. The ratio $R_{T_{2}}^{H_{2}}$ of resource $H_{2}$ in period $T_{2}$ is 100.0%. Then, the tight supply and shrinking supply–demand ratio occur with $R_{T_{1}}^{H_{1}}$, $R_{T_{2}}^{H_{1}}$, and $R_{T_{2}}^{H_{2}}$ updated to 62.5%, 79.2%, and 83.3%, respectively.

**Figure 9:**
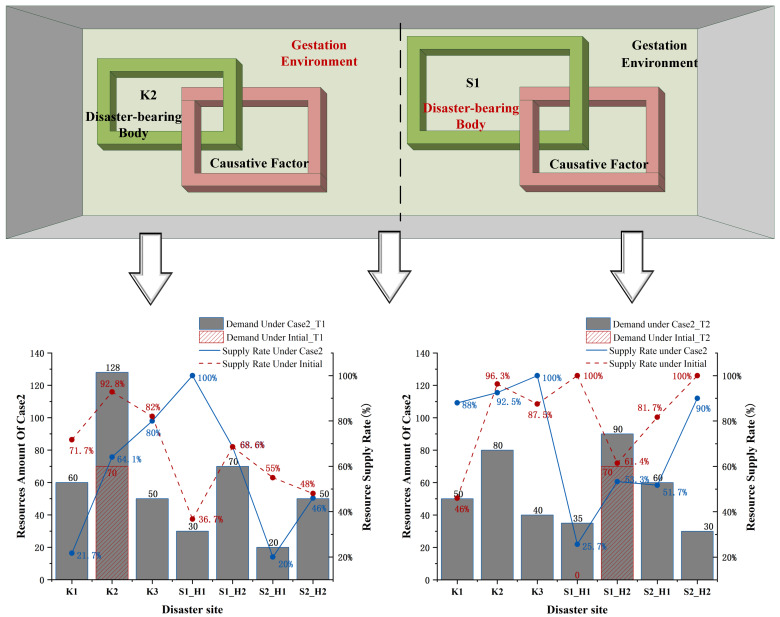
Allocation of emergency resource under the initial scenario and the case.

Comparing the results of emergency resource allocation under the scenarios, the supply increases to a certain extent when the proportion of demand increases at the corresponding disaster site. The total supply increases by 2.6% over the original, yet its overall unmet demand increases by 8.84% with a slight rise in travel time of 1.6%. As shown in Figure 9, the great majority of initial resource supply rates are higher than those after disaster amplification. When supply and demand are adjusted, resources tilt toward units with high demand and low distribution time costs. Nevertheless, this cannot address the sudden surge in relief needs and may further create imbalances in distribution rates across sites, ultimately leading to a social problem of regional inequity.

#### 4.2.4. Political Intervention in Disaster Chain Disposal

In the events of supply–demand imbalance or disaster coupling amplification, as simulated above, intervention mechanisms are needed to bring them back into balance. Ultimately, political intervention can generate informative solutions, as disposal of disasters are political spaces [53]. This section examines the role government intervention plays and how some risks can be eliminated through what additional resource capacity.

The government’s newly assigned budget is maximized for its effectiveness. In the meantime, relief fairness, specifically regional allocation rates, is also an important factor of government regulation that decision makers should have to take into account. Government departments increase the supply of corresponding emergency resources by setting up additional warehouses at suitable locations. The objective expression of minimizing the maximum demand unsatisfied rate is introduced to measure the regional allocation fairness, as defined by Equation (16). As can be observed from Table 4, these initiatives promote supply in general and balance the distribution of social resources in a local sense.

(16) $$Min\left(Max\left(U_{k}^{h,t},U_{s}^{h,t}\right)\right),k\in K,s\in S,h\in H,t\in T$$

**Figure 10:**
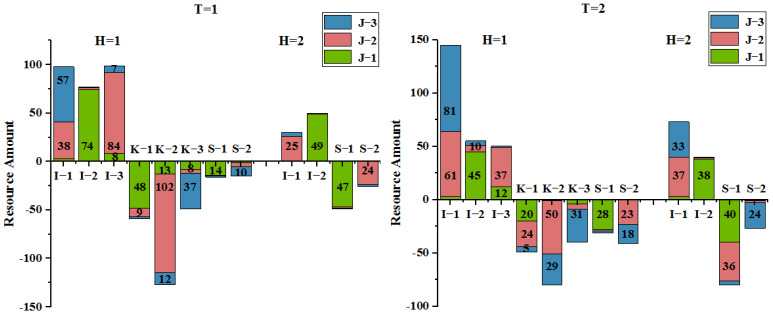
The resource flows of optimal rescue scheme.

Figure 10 displays the resource availability during the rescue process. The demand surge for emergency resource $H_{1}$ from PDP $K_{1}$ is supported by additional WH $I_{3}$ in period T=1. The burst resources demand for $H_{1}$ and $H_{2}$ from SDP $S_{1}$ is supported by WH $I_{1}$ with supplementary resource in period T=2. The supply is balanced and the total supply increases by 22.2%. The allocation of emergency resources under the natural disaster chain is a complicated decision with periodicity and fluctuation. Therefore, scientific policy support and modeling methods are necessary to provide decision makers with the optimal allocation scheme.

**Figure 11:**
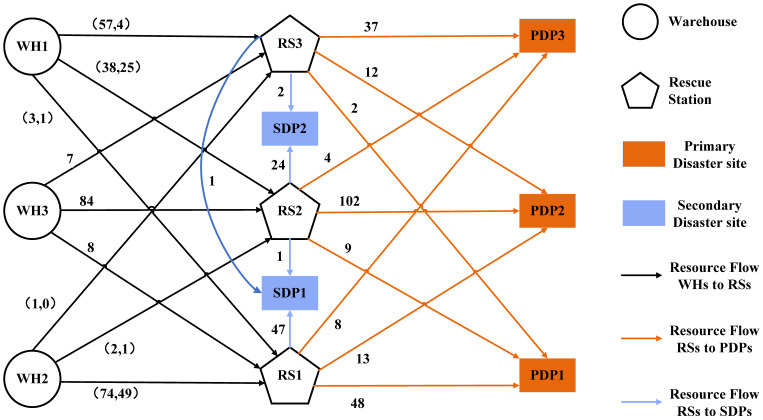
Optimized delivery flow network with additional resources in *T* = 1.

As can be observed intuitively from the delivery flow displayed in Figure 11, there is potential regional coordination in the optimal emergency resource allocation decision. Under the multiobjective constraint, warehouses, rescue stations, and disaster sites are matched with each other. For example, there are three main supply chains of “WH $I_{1}$—RS $J_{3}$—PDP $K_{3}$”, “WH $I_{2}$—RS $J_{1}$—PDP $K_{1}$”, and “WH $I_{3}$—RS $J_{2}$—PDP $K_{2}$”. From the perspective of government managers and logistics operators, this section has the following two implications. On the one hand, government managers can further divide the relief blocks and coordinate cross-regional transportation. Through the collaboration of logistics facilities and multiple supply routes, a positive circulation between regions can be formed and the normal supply of living materials can be ensured. On the other hand, the logistics operators in the collaborative distribution pattern can utilize the tense transportation resources. Reducing the “marginal cost” of cross-regional transportation through service sharing is especially important in the middle and late stages of relief.

## 5. Discussion and Conclusions

A multiobjective emergency resource allocation problem under the natural disaster chain was presented from methodology and operational research. The key scientific issues in disaster chain response were realized, such as dynamic response system, multilevel resource coordination, and emergency auxiliary decision. The resource allocation was creatively combined with path planning through the proposed MOCGA and improved A* algorithm with the raster map. MOCGA was applied to achieve parallel optimization of timeliness, efficiency, and fairness in humanitarian relief. The A* algorithm was improved to visualize and intellectualize the resource flow and maintain the trip reliability. The proposed model and algorithm can provide the optimal and scientific solution for emergency resource allocation in response to the natural disaster chain, coping with the uncertainty in rescue.

To further explore the identification and elimination of actual risks faced by decision makers, numerical studies were conducted under scenarios of logistics performance, coupling of disaster factors, and government regulation. When an important transportation hub in the original three-echelon logistics network fails, the efficiency of resource availability is greatly reduced. Thus, the overall performance of the scheme is affected by a 16.1% increase in unmet demand. Besides, the coupling of the causative factors chain, gestation environment, and disaster-bearing body in the disaster chain system brings additional resource allocation fluctuations. The overall unmet demand increases by 8.84% with a slight rise in travel time of 1.6%. If the supply cannot be kept up in time, it will trigger a serious break in the disaster relief loop. While neglecting regional distribution balance, the pursuit of resource supply efficiency at the macro level is also detached from the principle of postdisaster relief. In the subsequent analysis, the role of government regulation is added and reflects the fairness pursued by society. The government departments respond in real time and reallocate additional social resources as the disaster situation develops. The supply is balanced and the total supply rate increases by 22.2%. Therefore, the derivation of disaster situations is controlled by supplemental social resources, and forms potential regional coordination.

Disruption in infrastructure support, cascading effect of disasters, and time urgency are challenges imposed by the environment. The results show that the interconnection of A* algorithms and MOCGA is suitable and practically usable. The critical transportation hub derived by the A* algorithm and resilient supply mechanism provided by MOCGA play a key role in risk avoidance and resource availability. Vehicle path planning is the prerequisite and emergency resource allocation is the goal, thus forming a closed loop for the response of the actual natural disaster chain. Resilient infrastructure and supply schemes shift from remediation to preparedness, contributing significantly to overcome environmental damage. In this way, resilient and sustainable regions are formed in disaster prevention. The potential coordination of regional facilities and supply routes can be quite beneficial to both managers and operators in creating a virtuous circle between regions and reducing the “marginal cost” of cross-regional transport. In conclusion, the optimal allocation solution complemented by political intervention is a new working idea for scientific response of the disaster chain.

On future work, the study will be extended to other safety fields of the disaster chain and multiple transportation modes will be considered. The potential regional coordination identified in this paper could provide a basis for the next study on emergency response regarding biological disasters with regional sanitary barriers. Specific resource allocation strategies can also be relevant as future work.

## Figures and Tables

**Table 1:** The distances between WHs, RSs, PDPs, and SDPs.

	$I_{1}\;$	$I_{2}\;$	$K_{1}$	$K_{2}$	$K_{3}$	$S_{1}$	$S_{2}$
$J_{1}$	50	29	35	37	45	18	41
$J_{2}$	24	30	43	35	39	32	22
$J_{3}$	22	47	55	42	35	47	12

Notes: *J*_1_, *J*_2_, *J*_3_ represent the rescue stations; *I*_1_, *I*_2_ represent the warehouses; *K*_1_, *K*_2_, *K*_3_ represent the primary disaster points; *S*_1_, *S*_2_ represent the secondary disaster points.

**Table 2:** Supply of WHs in different periods.

	$H_{1}\;$		$H_{2}$	
	$I_{1}$	$I_{2}$	$I_{1}$	$I_{2}$
T=1	(90,100,110)	(75,80,95)	(25,30,35)	(55,60,65)
T=2	(130,150,170)	(50,60,70)	(40,50,60)	(50,50,50)

**Table 3:** Demand of PDPs and SDPs in different periods.

	$H_{1}\;$					$H_{2}$	
	$K_{1}$	$K_{2}$	$K_{3}$	$S_{1}$	$S_{2}$	$S_{1}$	$S_{2}$
T=1	(55,60,65)	(60,70,80)	(45,50,55)	(25,30,35)	(15,20,25)	(65,70,75)	(45,50,55)
T=2	(40,50,60)	(75,80,85)	(35,40,45)	(0,0,0)	(50,60,70)	(65,70,75)	(25,30,35)

**Table 4:** Allocation of emergency resource under changes in supply and demand ratios.

Disaster Sites	$K_{1}$		$K_{2}$		$K_{3}$		$S_{1}$		$S_{2}$	
Period	$T_{1}$	$T_{2}$	$T_{1}$	$T_{2}$	$T_{1}$	$T_{2}$	$T_{1}$	$T_{2}$	$T_{1}$	$T_{2}$
Demand/H1	60	50	70 → 128	80	50	40	30	0 → 35	20	60
Supply/H1	59	49	127	80	49	40	16	31	15	41
Demand/H2	/	/	/	/	/	/	70	70 → 90	50	30
Supply/H2	/	/	/	/	/	/	49	80	26	27

## Data Availability

The data presented in this study are available upon request from the corresponding author.
